# Osteoarthritis: A Review of Novel Treatments and Drug Targets

**DOI:** 10.7759/cureus.20026

**Published:** 2021-11-30

**Authors:** Mohini Panikkar, Elizabeth Attia, Sara Dardak

**Affiliations:** 1 Trauma and Orthopaedics, Royal Shrewsbury Hospital, Shrewsbury, GBR; 2 Stroke Medicine, University Hospital of Wales, Cardiff, GBR; 3 Trauma and Orthopaedics, St George's Hospital, London, GBR

**Keywords:** intervention, rehabilitation, orthopaedic, joint, osteoarthritis

## Abstract

Osteoarthritis affects over 10% of our population over the age of 60 years old, significantly reducing their quality of life and increasing morbidity. A number of aetiological factors contribute to the development of osteoarthritis including obesity, genetic factors, injury and increasing age. Many of the pathological processes which underlie the condition remain poorly understood and therefore limited progress has been made in developing effective disease modifying treatments. This review article aims to summarise our current understanding of osteoarthritis, the molecular mechanisms which drive the disease and current progress in developing therapeutic strategies to target these.

## Introduction and background

Osteoarthritis is the most common form of joint disease in developed societies, affecting over 10% of the population over the age of 60 years [1]. This disease conveys a substantial societal burden, and is associated with multimorbidity, disability and increased mortality [2,3]. Corresponding to the increase in life expectancy, the prevalence of this disease is projected to rise, with important global implications [1]. The recognition of the seriousness of this disease has driven the therapeutic agenda forwards, leading to intensified focus from academia and industry in recent years [4]. However, there remains relatively limited progress in the development of new treatments, with the lack of any disease-modifying drugs a pertinent issue [5]. This review aims to provide a summary and discussion of potential treatments and drug targets in osteoarthritis.

## Review

Pathogenesis

Osteoarthritis has previously been considered a disease of “wear and tear”, leading to the loss of articular cartilage [6]. However, progress in basic science and clinical research has shaped our understanding of the underlying pathogenesis, such that we now recognise this to be a mechanically driven disease involving the cartilage, synovium and subchondral bone [5]. This is compellingly presented in the epidemiological literature and supported by data from in-vitro systems and pre-clinical models [7,8]. It is evident that articular cartilage is highly mechanosensitive, with mechanical injury activating the inflammatory cascade and induction of proteases that initiate cartilage breakdown [8,9]. Factors such as age, obesity and genetics play a contributory role through their influence on the ability of the joint tissues to resist and repair damage caused by mechanical stress [5]. 

Potential treatments for osteoarthritis

Treatments Targeting Articular Cartilage

At a molecular level, one of the hallmarks of osteoarthritis is the dysregulation of anabolic and catabolic pathway enzymes in articular cartilage [10]. The identification of these pathways highlight the potential for disease-modifying drugs, with anabolic or anti-catabolic cartilage properties. 

Fibroblast growth factors (FGFs) have anabolic properties relating to the induction of chondrocyte proliferation and production of cartilage matrix. Sprifermin is an analogue of human FGF18, which has shown the most promise in clinical studies. In the Phase Ib trial, intra-articular injections of sprifermin in patients with symptomatic knee osteoarthritis demonstrated a significant dose-dependent reduction in loss of articular cartilage thickness compared to the placebo after 12 months [11]. 

In the subsequent Phase II trial (FORWARD), a significant improvement in articular cartilage thickness with intra-articular sprifermin compared to the placebo was reported after 24 months [12]. While these studies do not specifically demonstrate reversal of cartilage damage, they show that the damage can be arrested, which is promising. However, there is reason for caution. No differences in pain and function were observed between the two groups, thus, highlighting the uncertainty in the clinical significance of these findings. 

Matrix metalloproteinases (MMPs) are key proteases involved in the cartilage breakdown and are therefore favourable drug targets. However, the results of these thus far have been disappointing. Notably, a Phase II trial of the MMP inhibitor PG-116800 (PG-530742) was prematurely terminated due to musculoskeletal toxicity and no evidence of clinical benefit [13]. It has been postulated that the lack of specificity of this drug, which meant that it demonstrated affinity to a wide range of MMPs was a key contributing factor behind the adverse events [14]. Newer, more selective MMP inhibitors are currently under investigation, with no clinical data available yet [15].

Treatments Targeting Inflammatory Pathways

Inflammatory pathways within the joint have an important role in mediating the destruction of articular cartilage. Pre-clinical models of osteoarthritis have demonstrated the release of various pro-inflammatory mediators such as prostaglandins, cytokines and chemokines [6]. A plethora of drug targets exists, yet successful drugs have yet to materialise, likely due to redundancies within these pathways and the ubiquitous nature of these mediators. 

Interleukin (IL)-1 has an important role to play in mediating activation of MMPs and therefore, the transition from anabolic to catabolic pathway enzymes within the joint. Several drugs targeting the IL-1 receptor have been investigated with varying results. A Phase II trial comparing intra-articular anakinra injections (recombinant modified human IL-1 receptor antagonist protein) to a placebo showed similar improvements in the pain at as early as four days post-injection up till four weeks [16]. 

Another Phase II trial evaluated the role of AMG-108 (a fully human, immunoglobulin G2 monoclonal antibody against IL-1 receptor type 1) [17]. In this study, patients who received AMG 108 had greater, albeit non-significant improvement in pain compared to the placebo. An interesting finding was that patients with a higher baseline level of pain experienced greater improvement in pain. However, there is concern regarding the safety profile of AMG 108, which was found to decrease the neutrophil count and may have significant clinical implications. 

Similarly, drugs targeting tumour necrosis factor (TNF) have failed to demonstrate any efficacy in treating osteoarthritis. In a Phase II trial (EHOA) of etanercept for hand osteoarthritis, no meaningful benefit on pain was observed at the end of six months [18]. Findings for these suggest that the cytokine-mediated inflammation involved in osteoarthritis may not be central to the cause of the pain or cartilage damage in osteoarthritis [5]. 

Potential Drugs or Drug Targets

Several potential drug targets have been put forward based on pre-clinical data. Targeting proteases, which breakdown articular cartilage remains an attractive option despite yielding minimal success. One such protease, ADAMTS-5 has been identified as a principal aggrecan-degrading enzyme [19]. In the rat model, the combination of an ADAMTS-5 inhibitor (114810) and hyaluronic acid hydrogel ameliorated cartilage degeneration and promoted cartilage regeneration [20]. A recent Phase I trial of a small molecule inhibitor of ADAMTS-5 demonstrated good safety profile and evidence of target engagement, and is currently in the Phase II stage [21]. 

The Wnt family of signalling molecules are involved in a broad range of cellular responses, especially with regards to bone development. Mechanical stress on articular cartilage activates these molecules, which drive the induction of catabolic enzymes [22]. The use of a Wnt inhibitor (SM04690) in the murine model of osteoarthritis has shown disease-modifying potential of targeting this pathway [23]. The Phase I trial of intra-articular SM04690 injections reported acceptable safety, with further studies underway at present [24].

Vitamin A derivatives including all-trans-retinoic acid (ATRA) have an established role during skeletal development, with profound influences on the chondrocyte phenotype [25-27]. In vitro work has suggested increased signalling via the retinoid receptors in osteoarthritis [28]. This aligns with genome-wide association study (GWAS) data, which has implicated the retinoic acid pathway in the pathogenesis of hand osteoarthritis [29]. It has been postulated that the predilection of hand osteoarthritis in peri-menopausal women is due to the complex interactions between ATRA and oestrogen [30].

Discussion

At present, clinical success points towards targets involved in the anabolic pathway of enzymes, with drugs such as sprifermin demonstrating the most promise as a potential new treatment. In contrast, targets within the inflammatory pathway have not conferred significant yield. This is consistent with pre-clinical models, where GWAS in osteoarthritis suggest that a failure of repair is central to the pathogenesis of the disease. In particular, there is a notable absence of loci that predict the regulation of classical inflammatory genes in the GWAS models. 

This highlights an important issue regarding the role of inflammation in the pathogenesis of osteoarthritis, which has yet to be fully elucidated. Crucially, this is a question that needs to be answered to enable further progress in the identification and development of new drugs and drug targets. Efforts have been made to classify patients according to specific phenotypes, which is limited by the lack of cohesion between and within phenotypes. It is perhaps likely that osteoarthritis as a disease exists as a continuum rather than distinct phenotypes. The progress of large-scale molecular endotyping of patient samples may yield answers to these questions.

## Conclusions

Our continued progress in osteoarthritis research provides many reasons for optimism. The recognition of the complex interplay between signalling pathways within articular cartilage, synovium and subchondral bone has led to the identification of potential mechanistic drug targets. Given the multiple pathways involved in this disease, it is unlikely that targeting a single molecule by a specific mechanism will be effective at combating the disease. As with other chronic disorders, the future of osteoarthritis treatment may lie in combination therapy.
